# What You Should Eat

**Published:** 1885-03

**Authors:** 


### WHAT YOU SHOULD EAT.

Prof. Arnold says the great majority of people pay no attention to their health or mental sanity; wealth and distinction occupy their whole time and energy. They leave their health to the doctor, their quarrels to the lawyer, and their souls to the minister. But publications on health were now becoming more widely diffused and more broadly appreciated than formelry. The conditions in towns and cities were more favorable to the spread of disease than in rural localities. Some foods contained an excess of some constituents of the body; other foods contained a deficiency. Some foods were too heating; others were too cooling; some contained infection, pavinig the way for disease. Most all our ills can be traced to what goes into our mouths. The milk of every dam was a perfect food for her young. Human and bovine milks differed but little in their chemical composition, so the one could largely be substituted for the other.

But milk was not a perfect food for animals of advanced age. It stood to reason that after the period of growth was completed, the animal then required less mineral matter or bone forming material, and less nitrogenous or muscle forming material; only enough of these substances to repair the waste of the system was necessary. The compounds in the food which chiefly built muscular tissue were termed albuminoids; those making bone were mineral matters or ash; and those which supplied heat and formed fat were divided into fats and carbo-hydrates. All these constituents mnst exist in the food in the proper proportion during the different stages of animal growth, maturity and decay; and if the necessary proportions could not be found in one article of food, then the diet must be of a mixed nature. If the food contained too much fat or heat producing substance, the surplus must work off in some way, and so overtaxed some of the organs. So it also was, if the food contained too much nitrogenous matter or albuminoids, inducing a predisposition to kidney complaints, Bright's disease, etc. Milk was more nutritious than beef, and was not half so expensive, but it was too liquid for adults. This difficulty was overcome by condensing the milk. The milk of some breeds contained too much fat for human consumption; that of others contained too little, so a mixture would be about right. Milk was the result of a decomposition of tissue; and it was necessary that the cow should be healthy, and her food should not be of a stimulating nature. Filtering through charcoal would rid the milk of bad odors. In speaking of cream as an article of diet, the professor said that its chief richness lay in the portion which formed the butter-milk, but the virtue of the butter fats consisted in the fat being in a more digestible form than other fats. The volatile oils of the cream being attenuated, were readily available, and so the cream was good for invalids, and was the best cod-liver oil that could be procured. Butter was a pure luxury; cream was both a luxury and a necessary, and was an excellent brain food, being more of a lubricant than a stimulant for the brain. Butter was a wasteful and worthless product. For a few cents, science could replace everythlng that was contained in a whole pound of butter. A little education might prevent people from sacrificing nine-tenths of the milk in order that one-tenth in the form of a luxury should be saved; but'there was money in it, and there the matter ended. The cheese was the bone and sinew of the milk.—*Farmers' Advocate.*

